# Feasibility, Adherence and Efficacy of Liraglutide Treatment in a Sample of Individuals With Mood Disorders and Obesity

**DOI:** 10.3389/fpsyt.2018.00784

**Published:** 2019-01-23

**Authors:** Alessandro Cuomo, Simone Bolognesi, Arianna Goracci, Cristina Ciuoli, Bruno Beccarini Crescenzi, Giuseppe Maina, Gianluca Rosso, Edvige Facchi, Carla Maccora, Nicola Giordano, Valeria Verdino, Andrea Fagiolini

**Affiliations:** ^1^Department of Molecular and Developmental Medicine, University of Siena, Siena, Italy; ^2^Department of Neuroscience Rita Levi Montalcini, University of Turin, Turin, Italy; ^3^Azienda USL Toscana Sud Est, Arezzo, Italy

**Keywords:** liraglutide, mood, obesity, bipolar, depression, depressive disorder

## Abstract

**Background:** Liraglutide is a once-daily injectable medication approved for the treatment of obesity. Hereby we report the feasibility, adherence and efficacy of liraglutide treatment in a sample of individuals with mood disorders and obesity.

**Methods and Sample:** Twenty-nine patients with Bipolar or Major Depressive Disorder received liraglutide once daily subcutaneously at a dose gradually titrated from 0.6 to 3 mg. All patients were obese and had previously failed multiple healthy lifestyle interventions, including exercise and diet programs. Patients' weight was recorded before liraglutide treatment (T0) and then 1 (T1), 3 (T3), and 6 months (T6) following T0.

**Results:** Mean baseline (T0) weight was 110.54 Kg (±24.95). Compared to baseline, the percentage of weight loss was 3.37% at T1, 7.85% at T3, and 10.20% at T6. Thirty-one percent (*n* = 9) of patients had no side effects, 34.48% (*n* = 10) had one, 24.14% (*n* = 7) had two, and 10.34% (*n* = 3) had three side effects. All 29 subjects were still on liraglutide at T1; 79.31 and 48.28% were on liraglutide at T3 and T6. No significant relationship was found between liraglutide dose and likelihood to continue the medication. No patient showed a worsening of the psychiatric condition due to liraglutide treatment. Acceptability and satisfaction with treatment were good for the 48% that completed the study.

**Conclusions:** Liraglutide treatment was efficacious, accepted and tolerated by ~50% of our sample, followed up for a period of 180 days. Larger, longer, controlled, prospective studies are warranted.

## Introduction

Several studies have pointed to a link between cardiovascular diseases, obesity or metabolic syndrome among patients suffering from severe psychiatric disorders (1).

For instance, patients suffering from depression, bipolar disorder and schizophrenia have 53% higher chance of cardiovascular disease, comparing to persons without psychiatric disorders, and their life expectancy is decreased by at least one or two decades (2). In addition, Weiner and associates found that the risk of cardiovascular mortality is twice as larger among persons with diagnosed bipolar disorder compared to general population (3).

The high prevalence of obesity, metabolic syndrome and cardiovascular disease among patients diagnosed with major psychiatric disorders may be a result of genetic factors, inadequate lifestyles, such as poor dietary habits, lack of physical activity and tobacco smoking, adverse effects of psychotropic drugs, and environmental factor (4). Patients with psychiatric diseases may also have poorer access to care, which may affect the prevalence and severity of physical illnesses (5).

Liraglutide is an agonist of the acylated human Glucagon-Like-Peptide-1 (GLP-1) receptor with 97% of sequence homology with naturally produced GLP-1. This medication is the first once-daily injectable derivative of the human incretin glucagon-like peptide-1 recently approved by the European Medicines Agency (EMA) and the United States Food and Drug Administration (FDA) for the treatment of obesity (adults with a BMI ≥ 30 kg/m^2^), as well for adults with a BMI of 27 kg/m^2^ or greater who also have one or more complications related to their weight, such as type 2 diabetes, high blood pressure, high cholesterol or obstructive sleep apnea (6, 7). Liraglutide reduces body weight as well fatty mass through mechanisms that decrease the need for food and energy intake. This agent also improves glycemic control by reducing sugar levels in patients with type 2 diabetes mellitus (8). Liraglutide stimulates the secretion of insulin and reduces the excessive elimination of glucagon, based on glucose concentration. When glucose level is high, insulin release is stimulated and glucagon release inhibited. Conversely, during hypoglycemia, liraglutide decreases the release of insulin, without affecting glucagon release.

We recently reported on the development, acceptability and efficacy of a standardized healthy lifestyle intervention in recurrent unipolar and bipolar depression (9). Our lifestyle intervention includes modules dealing with energy balance, physical exercise, nutritional education and other strategies to lose weight with physical exercise and to not relapse into bad eating habits and sedentary life. Among other findings, we observed a significant reduction in BMI in patients who received the intervention, compared to controls. However, while conducting the trial above, and during our daily practice, we observed that–for a considerable number of patients- a lifestyle behavioral intervention may not be sufficient to achieve the necessary improvement in metabolic parameters. To this end, we decided to test the feasibility, adherence, weight effects and tolerability of liraglutide treatment, administered for a period of 6 months in a sample of patients with mood disorders.

## Materials and Methods

This was a retrospective study involving individuals with bipolar or major depressive disorder who received liraglutide. All study participants had previously failed multiple standardized healthy lifestyle interventions, including physical exercise and diet programs. Patients weight and BMI were registered at four different time-points: baseline, i.e., when liraglutide was started (T0), and then after 1 (T1), 3 (T2), and 6 months (T3).

Data were analyzed using STATA15 (Stat Corp., College Station, Texas, USA). Descriptive statistics, Wilcoxon signed-rank test and Fisher exact test were performed. The statistical significance was set at level of 5% (*p* < 0.05). All study data was collected retrospectively and no exam was specifically conducted for the purposes of this study. For the reasons previously mentioned, the study was exempt from informed consent. The retrospective study was approved by the Regione Toscana–Area Vasta Sud Est Ethical Committee Board.

## Results

### Sample Characteristics

The sample included 29 patients diagnosed with mood disorders and treated at University of Siena Medical Center, Italy. There were almost four times more female patients (F = 80% of the sample). Mean age was 53.48 ± 12.75 years and half of participants (51.72) were employed. Twenty-one percent were smokers. The vast majority was physically inactive and all participants were obese. Four patients were in treatment with second generation antipsychotics: 2 with risperidone, 1 with olanzapine and 1 with aripiprazole. Hyperlipidemia and systolic hypertension were the most frequent physical comorbidities (Table 1).

**Table 1:** Sample characteristics.

**Patients' sociodemographic characteristics**		**Frequency (%)**
Total number of participants		29
Gender	Female	23 (79.31)
	Male	6 (20.69)
Marital Status	Single	11 (37.93)
	Married	18 (62.07)
Educational Background	Primary/Middle School	8 (27.59)
	High School Diploma	13 (44.83)
	Bachelor	8 (27.59)
**PATIENTS ′ LIFESTYLE**		
Smoking tobacco		6 (20.69)
Physical activity		5 (17.24)
Diet	Between meals	23 (79.31)
	At meal time	6 (20.69)
**PATIENTS ′ BMI**		
Obesity Class I (BMI 30–34, 9 kg/m^2^)		4 (13,79)
Obesity Class II (BMI 35–39, 9 kg/m^2^)		25 (86,21)
**PATIENTS ′ COMORBIDITIES**		
Type 2 diabetes		6 (20.69)
Impaired glucose tolerance		7 (24.14)
Hyperlipidemia		16 (55.17)
Hypertension		13 (44.83)

### Weight and BMI

At T1 (i.e., 1 month after liraglutide was started), a significant weight loss was observed (*p* < 0.001) and the mean weight decreased from 110.54 (±24.95) kg at baseline (T0) to 106.81 (±24.43) kg at T1 (Table 2). On average, patients lost 3.37% of their baseline weight. Only two patients did not lose weight in the first 1 month of treatment, and they both decided to discontinue liraglutide. At the 3-month time point (T3), the weight loss compared to baseline (T0) remained significantly lower (*p* = 0.0001). Mean weight at T3 was (101.84 ± 17.92 kg) (Table 2). On average, subjects lost 7.85% of their baseline weight. Six patients discontinued liraglutide because of the following reasons: pancreatic enzymes (lipase and amylase) increase (*n* = 1), bariatric surgery (*n* = 1), no declared reason (*n* = 1), treatment ineffectiveness (*n* = 2); lost to follow up (*n* = 1).

**Table 2:** Weight, weight loss (%), BMI and BMI loss (%) of patients at T0, T1, T3, and T6.

	**N**	**Average weight ± SD (kg)**	**%**	***p***
Baseline	29	110.54 (±24.95)	–	–
1 month (T1)	29	106.81 (±24.43)	−3.37	*p* < 0.01
3 months (T3)	23	101.84 (±17.92)	−7.85	*p* < 0.01
6 months (T6)	14	99.26 (±20.53)	−10.20	*p* < 0.01
	**N**	**Average BMI ± SD (kg/m**^**2**^**)**	**%**	
Baseline	29	41.28 (±7.63)	–	–
1 month (T1)	29	39.90 (±7.22)	−3.34	*p* < 0.01
3 months (T3)	23	40.65 (±11.43)	−1.53	*p* < 0.01
6 months (T6)	14	38.4 (±6.57)	−6.98	*p* < 0.01

*N, Number of patients*.

At the 6 months' time-point (T6) a significant weight loss compared to baseline (T0) (*p* = 0.0010) was recorded as well. Mean weight at T6 was 99.26 (±20.53) (Table 2). On average, patients lost 10.2% of their baseline weight. However, only 14 patients, out of the original 29, were still taking liraglutide. The reasons for discontinuation among 15 study participants who did not complete 90 day of treatment were primarily due to treatment ineffectiveness and/or undesirable side effects related with liraglutide use.

### Tolerability

The following undesirable side effects were recorded: amylase and lipase increase, nausea, vomiting, diarrhea, and constipation. Nine of the 29 patients (31%) reported no side effects, ten (34%) reported one side effect, seven (24.14%) reported two side effects, and 3 (10%) reported three side effects.

No significant relationship was observed between liraglutide dose and number or severity of side effects. Similarly, no relationship between physical comorbidities and side effects was noted.

## Discussion

To our knowledge, only few studies have evaluated the feasibility and safety of liraglutide treatment in patients with mood disorders. For instance, Mansour and colleagues evaluated the ability of liraglutide to improve cognitive function in 19 subjects with mood disorders and found that liraglutide was well-tolerated and had beneficial effects on cognitive function. The study was a 4-week pilot, open-label trial, and 17 of the 19 participating subjects completed the trial (10). In patients with schizophrenia spectrum, Larsen et al. (11) conducted a double-blind trial involving 103 overweight/obese individuals who were prediabetic. Study subjects were randomized to a 16-week treatment with either liraglutide or placebo, while receiving clozapine or olanzapine. Fifty-two individuals received liraglutide. Five of these patients did not complete the 16-week treatment, because of thyrotoxicosis (*n* = 1), worsening of psychiatric disorder (*n* = 2), discontinuation or dose reduction of olanzapine or clozapine (*n* = 2), death (*n* = 1). The Authors showed that liraglutide treatment administered for 16 weeks significantly reduced glucometabolic disturbances and body weight in overweight or obese and pre-diabetic patients with schizophrenia-spectrum disorders, receiving clozapine or olanzapine. Patients were then followed for 1 year after discontinuing liraglutide. Although body weight reduction was partially sustained, the improvements in other metabolic variables returned to the levels that had been recorded before starting the 16-week treatment with liraglutide (12). In our study, liraglutide was continued for at least 6 months and proved efficacious in about 50% of the study sample (14 out of 29) whereas the remaining 50% discontinued the medication before completing a 6 months treatment period, primarily because of inefficacy or side effects. Among the latter, nine participants asked to discontinue the medication (5 due to inefficacy; 4 due side effects), five dropped out without presenting at the following appointments, and one discontinued the treatment in agreement with the treating physician (because of bariatric surgery). The rate of persistence with liraglutide treatment was lower than the rate observed in liraglutide trials involving subjects that were not selected based on their psychiatric diagnosis (13, 14), this suggesting the possibility that patients with mood disorders may be less likely to adhere to liraglutide treatment, possibly because of their mental disorder. This rate was also lower than the trials in patients with mental illness mentioned above, which however were conducted in different settings and for different periods. Nonetheless, we find it interesting that ~50% of our sample was able to tolerate, continue and respond to liraglutide for a 6-month period. Neuropsychiatric safety of treatment with liraglutide was evaluated pooling data from the liraglutide weight-management programme, with no between-treatment imbalances being noted for depression or suicidal ideation/behavior (15). Consistently with this study, no neuropsychiatric side-effects nor any liraglutide related worsening of the pre-existing mental condition were noted in our study. However, our research has several limitations that should be acknowledged, including: (1) the small sample size, which clearly does not permit to consider our sample as representative of all patients with mood disorders; (2) the retrospective and non-randomized design; (3) the absence of a placebo-control group; (4) the relatively short duration (6 months) of the observation period. Larger, longer, controlled, prospective trials including measures of metabolic parameters, quality of life, daily functioning and course of the psychiatric disease are warranted.

## Author Contributions

AC, SB, and AG: contributed to study design, data collection, data analysis, data interpretation, and manuscript drafting. CC: contributed to study design, data collection, data interpretation, and manuscript drafting. BB, GM, GR, EF, CM, NG, VV, and AF: contributed to study design, data interpretation, and manuscript drafting.

### Conflict of Interest Statement

The authors declare that the research was conducted in the absence of any commercial or financial relationships that could be construed as a potential conflict of interest.
